# Epstein-Barr Virus BBLF1 Mediates Secretory Vesicle Transport to Facilitate Mature Virion Release

**DOI:** 10.1128/jvi.00437-23

**Published:** 2023-05-17

**Authors:** Md Kamal Uddin, Takahiro Watanabe, Masataka Arata, Yoshitaka Sato, Hiroshi Kimura, Takayuki Murata

**Affiliations:** a Department of Virology, Nagoya University Graduate School of Medicine, Nagoya, Aichi, Japan; b Precursory Research for Embryonic Science and Technology (PRESTO), Japan Science and Technology Agency (JST), Kawaguchi, Saitama, Japan; c Department of Virology and Parasitology, Fujita Health University School of Medicine, Toyoake, Aichi, Japan; University of Toronto

**Keywords:** EBV, BBLF1, lytic replication, viral release, secretory vesicles

## Abstract

Enveloped viruses undergo a complex multistep process of assembly, maturation, and release into the extracellular space utilizing host secretory machinery. Several studies of the herpesvirus subfamily have shown that secretory vesicles derived from the *trans*-Golgi network (TGN) or endosomes transport virions into the extracellular space. However, the regulatory mechanism underlying the release of Epstein-Barr virus, a human oncovirus, remains unclear. We demonstrate that disruption of BBLF1, a tegument component, suppressed viral release and resulted in the accumulation of viral particles on the inner side of the vesicular membrane. Organelle separation revealed the accumulation of infectious viruses in fractions containing vesicles derived from the TGN and late endosomes. Deficiency of an acidic amino acid cluster in BBLF1 reduced viral secretion. Moreover, truncational deletion of the C-terminal region of BBLF1 increased infectious virus production. These findings suggest that BBLF1 regulates the viral release pathway and reveal a new aspect of tegument protein function.

**IMPORTANCE** Several viruses have been linked to the development of cancer in humans. Epstein-Barr virus (EBV), the first identified human oncovirus, causes a wide range of cancers. Accumulating literature has demonstrated the role of viral reactivation in tumorigenesis. Elucidating the functions of viral lytic genes induced by reactivation, and the mechanisms of lytic infection, is essential to understanding pathogenesis. Progeny viral particles synthesized during lytic infection are released outside the cell after the assembly, maturation, and release steps, leading to further infection. Through functional analysis using BBLF1-knockout viruses, we demonstrated that BBLF1 promotes viral release. The acidic amino acid cluster in BBLF1 was also important for viral release. Conversely, mutants lacking the C terminus exhibited more efficient virus production, suggesting that BBLF1 is involved in the fine-tuning of progeny release during the EBV life cycle.

## INTRODUCTION

Epstein-Barr virus (EBV) is a double-stranded DNA virus belonging to the family *Herpesviridae* (1). EBV infects >95% of the global population, often leads to asymptomatic infections, and is involved in the development of various cancers. EBV infection is associated with epithelial cancers, including nasopharyngeal and gastric cancers, as well as B-cell, NK-cell, and T-cell lymphomas (2–5). Approximately 2% of all malignant tumors are caused by EBV infection, and more than 200,000 new cases of EBV-associated cancer are estimated to occur worldwide each year (6, 7).

There are two types of EBV infection as follows: latent and lytic. In general, EBV establishes a latent infection without viral production. The switch from latent to lytic infection is called reactivation, which leads to the production of viral particles. Latent cells can be reactivated *in vitro* by several chemical and biological stimuli, including 12-*O*-tetradecanoylphorbol-13-acetate, transforming growth factor β, histone deacetylase inhibitors, and anti-human IgG (8–10). Upon reactivation, cells express >80 viral genes, classified as immediate early (IE), early (E), and late (L) genes according to their cascade pattern (11). The expression of IE genes (BZLF1, and BRLF1) induces the expression of E genes involved in viral DNA replication, including BALF2, BMRF1, BBLF4, BSLF1, BBLF2/3, and BALF5 (12–15). Six E-class gene products, BcRF1, BDLF3.5, BGLF3, BFRF2, BDLF4, and BVRF1, activate the transcription of L genes as a viral preinitiation complex (vPIC) (16–18), resulting in the expression of proteins that comprise viral particles or play roles in their morphogenesis and release. The assembly and egress of herpesviruses have been most extensively studied using herpes simplex virus 1 (HSV-1), which is the prototype of *Herpesviridae*. The amplified viral genome is incorporated into icosahedral capsids in the nucleus, thereby forming nucleocapsids. Some tegument proteins, such as capsid-associated tegument complex (CATC) are attached to nucleocapsids in the nucleus. The nucleocapsids then exit the nucleus and migrate to the cytoplasm through the nuclear membrane via an envelopment and de-envelopment process. In the cytoplasm, the nucleocapsids bud into special vesicles derived from the *trans*-Golgi network (TGN), early endosome (EE), or autophagosome, where they acquire additional tegument proteins, the viral envelope, and glycoproteins (19). Eventually, the fusion of these vesicles with the plasma membrane causes the release of mature virions into the extracellular milieu (20–23). Previous studies of gammaherpesvirus have shown that lytic proteins enhance various stages of virion morphogenesis and egress. For example, the EBV-encoded protein kinase BGLF4 and nuclear egress complex BFRF1/BFLF2 modify nuclear membrane structure and promote the exit of nucleocapsids from the nucleus to the cytoplasm (24–26). The protein complex of BLRF1/BBRF3 (gN/gM) (27) and Kaposi's sarcoma-associated herpesvirus (KSHV) glycoprotein gB contributes to viral assembly and egress (28). Comprehensive analysis of an intraviral protein interactome showed that BLRF2 is a central hub of the tegument network, suggesting that BLRF2 is vital for efficient tegumentation (29). Mature EBV gains its final envelope in intracellular compartments rich in Golgi markers and exploits the host cell’s secretory pathway for release (30, 31).

The presence of tegument, a proteinaceous matrix between the nucleocapsid and envelope, is a hallmark of all herpesviruses. Tegument proteins play crucial roles in herpesvirus biology and pathogenicity, including the establishment of primary infection (32), modulation of the cellular environment (33), virion morphogenesis (34, 35), and immune evasion (36). Purified EBV particles contain at least 17 virus-encoded tegument proteins, including BBLF1 (37). BBLF1 is myristoylated and palmitoylated during posttranslational modification and contains two clusters of acidic amino acids (38). During the secondary envelopment process, both myristoylation and palmitoylation are required for stability and membrane anchorage of this protein. Meanwhile, the acidic clusters of BBLF1 interact with cellular phosphofurin acidic cluster sorting protein 1 (PACS-1), which plays a regulatory role in intracellular trafficking to the TGN membrane. Moreover, interactions between the KSHV proteins open reading frame 33 (ORF33) and ORF38 (28) and between the EBV proteins BGLF2 and BBLF1 (39) during the maturation stage are important for infectious virus production. Knockdown of BBLF1 in P3HR1 cells decreased virus production (38). Knockout BBLF1 homologs, namely, UL11 of HSV-1 and UL99 of human cytomegalovirus (HCMV), caused intracellular accumulation of progeny viruses (40, 41). Hence, herpesvirus tegument proteins exploit various cellular and viral proteins to facilitate virion production (42). However, the mechanism through which BBLF1 mediates virus exit during the lytic cycle remains unclear.

Here, to clarify the role of the BBLF1 gene product in virus production, we established a BBLF1-knockout virus and found that disruption of the BBLF1 gene led to an accumulation of virions within cells. Exogenous supply of BBLF1 to the knockout virus restored virion release. Transmission electron microscopy (TEM) and subcellular fractionation analysis showed that BBLF1 knockout led to an accumulation of virions in secretory vesicles. Through mutagenesis of BBLF1, we found that optimal shedding of virions required a cluster of acidic amino acids but did not require posttranslational modifications, such as myristoylation and palmitoylation of the protein. By contrast, truncation of the C-terminal region of BBLF1 increased infectious virus production, suggesting that the C terminus of BBLF1 regulates its production function.

## RESULTS

### BBLF1 is an EBV lytic gene expressed with late kinetics during the productive cycle.

**FIG 1:**
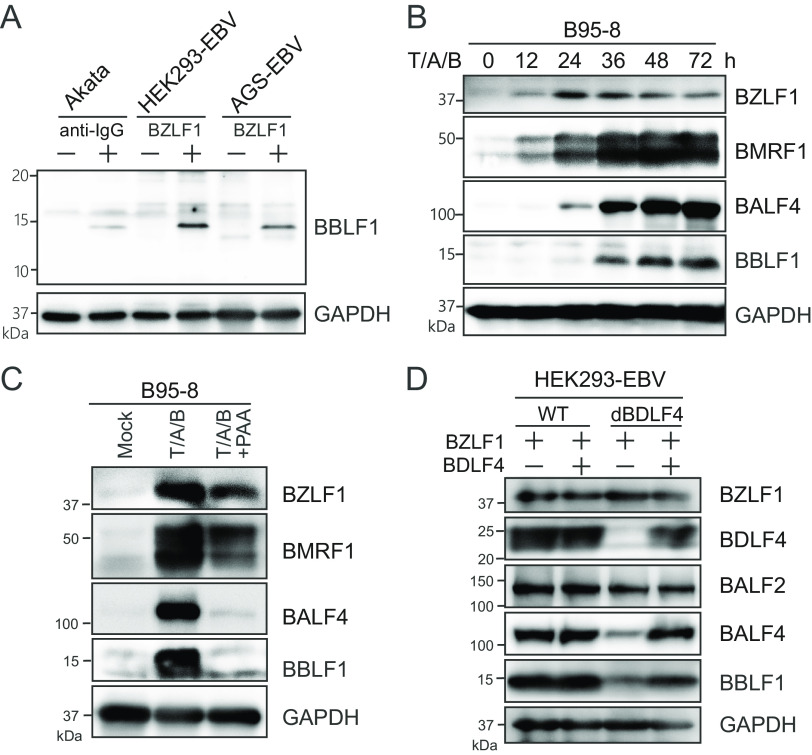
EBV BBLF1 is a late lytic protein. (A) Identification of endogenous BBLF1. Akata cells were treated with anti-human IgG antibody, and HEK293-EBV and AGS-EBV cells were transfected with the BZLF1 expression vector for lytic reactivation. Cells were harvested at 48 h after reactivation and then subjected to immunoblotting (IB) using a newly generated anti-BBLF1 antibody. (B) B95-8 cells were treated with T/A/B (12-*O*-tetradecanoylphorbol-13-acetate [TPA], A23187, and sodium butyrate) for lytic induction, collected at the indicated time points and monitored for viral protein expression via IB for BZLF1 (IE), BMRF1 (E), BALF4 (L), and BBLF1, with GAPDH as a housekeeping gene. (C) B95-8 cells were treated with T/A/B with or without phosphonoacetic acid (PAA), which is a viral DNA polymerase inhibitor. (D) HEK293 cells stably carrying the EBV WT or BDLF4 knockout viral genomes were lytically reactivated through BZLF1 transfection in the presence or absence of pBDLF4, harvested after 48 h, and subjected to IB with the indicated antibodies.

To detect endogenous BBLF1 protein during the lytic stage, we first generated a polyclonal antibody against a BBLF1 polypeptide in rabbits and then purified it through affinity chromatography. This antibody efficiently detected endogenous BBLF1 (molecular mass, approximately 15 kD) in lytically induced Akata, HEK293-EBV, and AGS-EBV cells through immunoblotting (Fig. 1A). To observe the time points of detectable BBLF1 expression, EBV-positive B cells reactivated by treatment with 12-*O*-tetradecanoylphorbol-13-acetate, calcium ionophore, and sodium butyrate were harvested sequentially. BBLF1 expression was detected 36 h after lytic reactivation, similar to the L-stage lytic protein BALF4 (Fig. 1B). When treated with phosphonoacetic acid, a viral DNA polymerase inhibitor, BBLF1 expression was strongly inhibited, similar to the L gene BALF4 (Fig. 1C). Knockout of BDLF4, an essential component of the vPIC that enhances L gene expression (17), suppressed the expression of BBLF1, while *trans*-BDLF4 expression complemented the decreased expression levels of both BBLF1 and BALF4 (Fig. 1D), suggesting that BBLF1 expression is dependent on the vPIC. These data indicate that the BBLF1 gene is expressed with L kinetics during the EBV production cycle.

### Construction of the BBLF1-knockout virus using the EBV bacterial artificial chromosome system.

**FIG 2:**
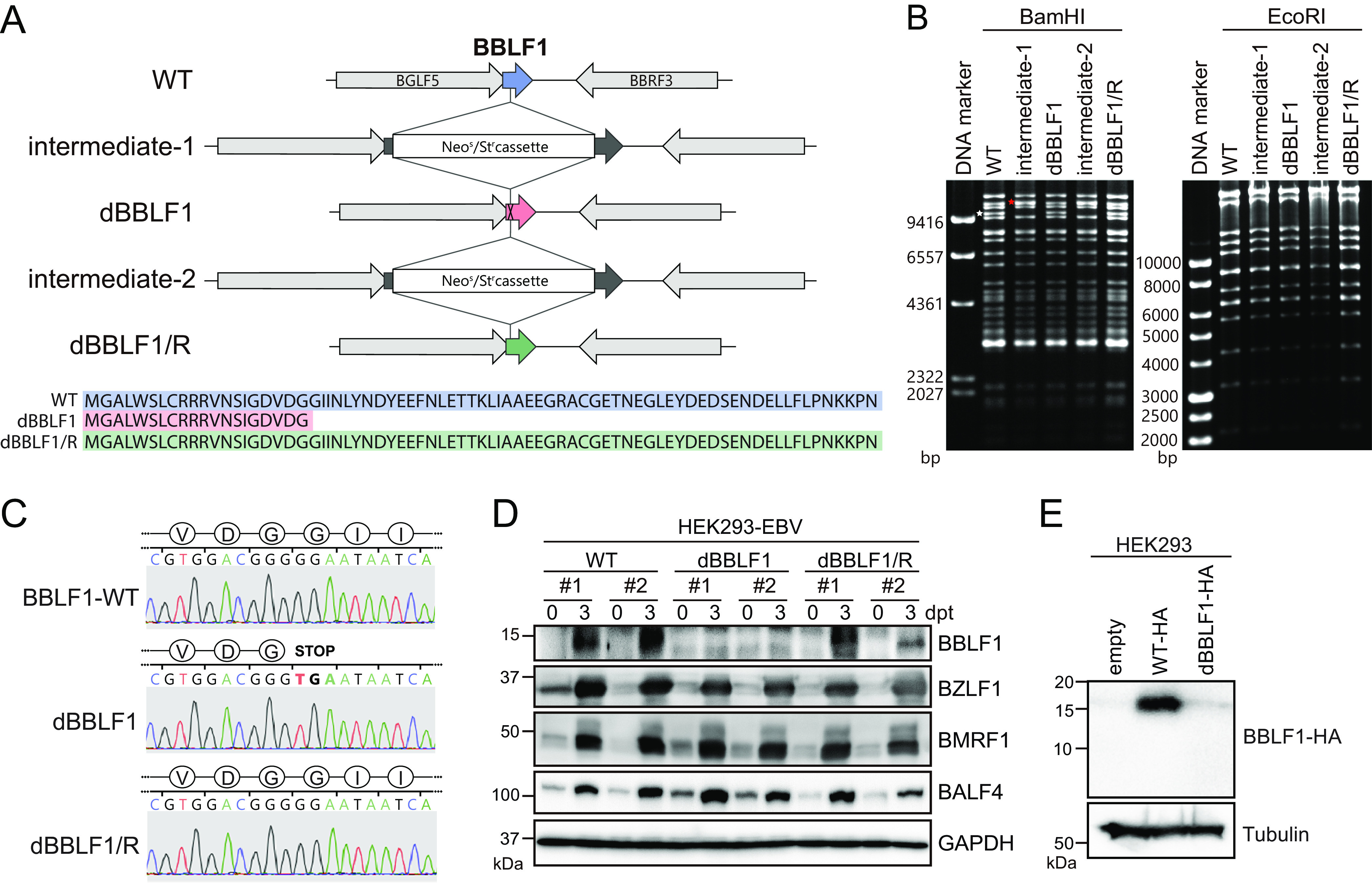
Generation of the BBLF1-knockout EBV mutant using the EBV-BAC system. (A) Diagram of the construction of BBLF1-knockout (dBBLF1) and -revertant (dBBLF1/R) viruses using the homologous recombination technique in E. coli. An intermediate of EBV-BAC (intermediate-1) was first created through insertion of a tandemly arranged neomycin-sensitive and streptomycin-resistance gene cassette (Neo^s^/St^r^) at the 61st nucleotide of the BBLF1 gene in the WT EBV-BAC. The cassette was then replaced with a sequence of BBLF1 carrying the G61 to T61 mutation, which introduces a stop codon into the BBLF1 gene. Similarly, a revertant virus (dBBLF1/R) was generated from the dBBLF1 virus with exactly the same genetic construct as the WT. (B) Electrophoresis of digested EBV-BAC DNA. Isolated EBV-BACs (WT, dBBLF1, and dBBLF1/R) were digested through treatment with BamHI or EcoRI and then electrophoresed. In the lane containing intermediates 1 and 2 (BamHI digests), the DNA fragment containing the BBLF1 gene (indicated with a white asterisk in the WT lane) migrated more slowly due to the size of the Neo^s^/St^r^ cassette (indicated with a red asterisk). (C) Nucleotide sequencing of EBV-BAC DNA for the generation of the BBLF1-knockout and revertant viruses. (D) Clonal HEK293-EBV cells (WT, dBBLF1, and dBBLF1/R) were collected immediately or at 3 days post-BZLF1 transfection (dpt). The indicated expression levels of EBV and GAPDH proteins were examined through IB. (E) Expression vectors carrying WT and dBBLF1 sequences with a C-terminal HA-tag were constructed and transfected into HEK293 cells. Cells were subjected to IB using anti-HA and anti-tubulin antibodies.

To determine the biological role of BBLF1, we generated a BBLF1-knockout virus (B95-8 strain) using the EBV bacterial artificial chromosome (BAC) system with a homologous recombination technique. A BBLF1-deficient virus (dBBLF1) carrying a stop codon (TGA) was generated from the wild type (WT), and a revertant virus (dBBLF1/R) was constructed from dBBLF1 (Fig. 2A). Because BBLF1 overlaps with the neighboring BGLF5, the stop codon in dBBLF1 was introduced at its 21st amino acid position to avoid the overlapping region. Isolated EBV-BAC DNA digested with BamHI and EcoRI was observed by agarose gel electrophoresis to verify the integrity of the viral genome (Fig. 2B). Sequencing analysis revealed that the expected mutant virus was generated successfully (Fig. 2C). Through transfection of EBV-BAC DNA (WT, dBBLF1, or dBBLF1/R) into HEK293 cells, we established clonal cell lines stably carrying EBV-BAC DNA. We then analyzed the expression levels of viral proteins during the lytic cycle. The viral proteins BZLF1, BMRF1, and BALF4, representative of the IE, E, and L stages of the lytic cycle, respectively, were measured along with BBLF1 (Fig. 2D). The expression of these viral proteins in knockout-infected cells was comparable to that in the WT and dBBLF1/R. These data indicate that BBLF1 knockout does not hamper the expression of representative viral proteins. Immunoblotting did not reveal BBLF1 expression in cells infected with the dBBLF1 virus because the epitope region of the antibody was lost (Fig. 2D). To determine if dBBLF1 was expressed, we transfected expression vectors carrying hemagglutinin (HA)-tagged dBBLF1 sequences into the parental strain (HEK293 cells). The expression of dBBLF1-HA (MGALWSLCRRRVNSIGDVDG-HA tag) was below the detection limit, indicating that it is extremely unstable (Fig. 2E). These data suggest that 20 N-terminal amino acids of BBLF1 are not functional.

### BBLF1 is involved in virion release.

**FIG 3:**
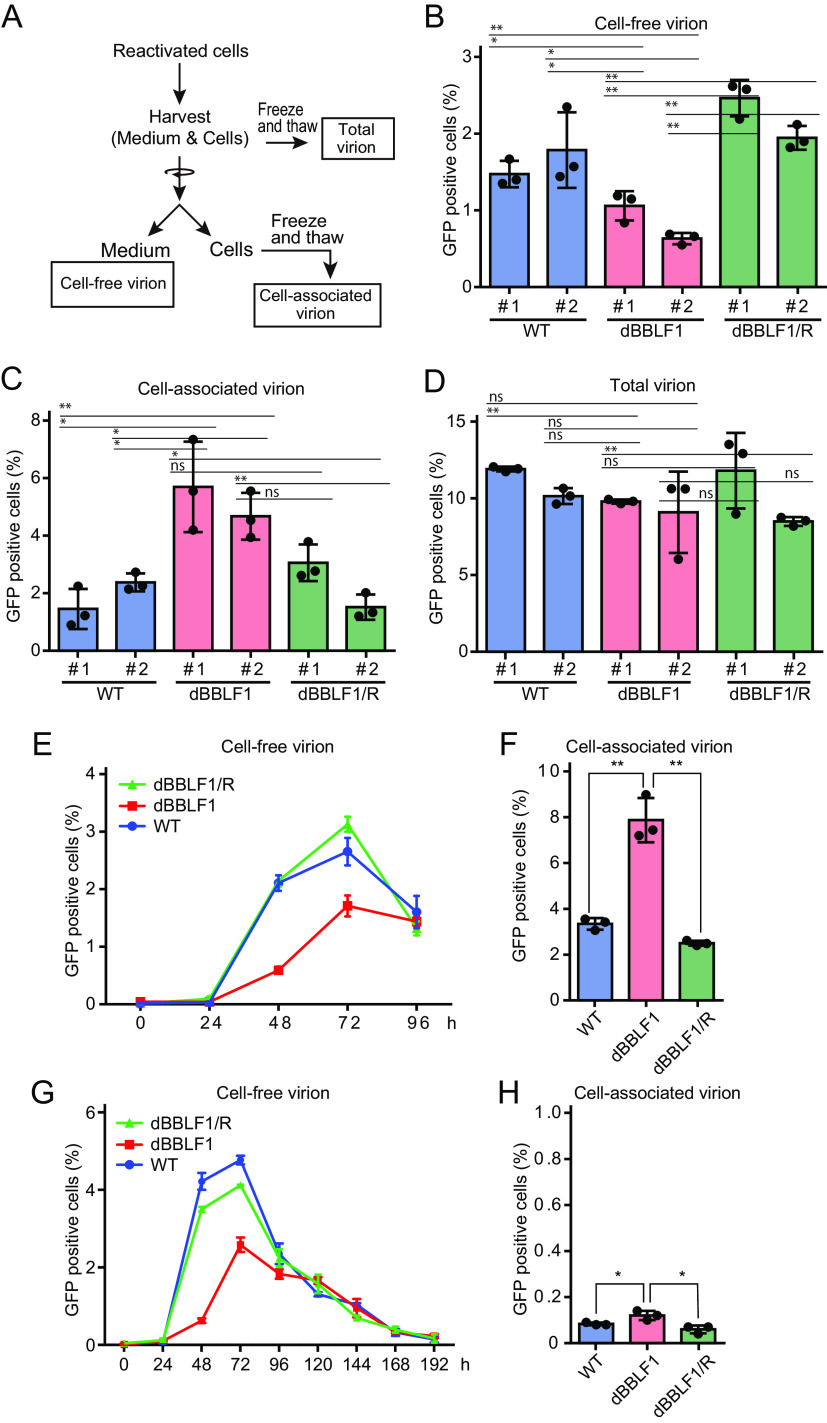
BBLF1 promotes virion release during lytic infection. (A) Virus samples of three fractions as follows: cell-free, cell-associated, and total. At day 3 after reactivation, HEK293-EBV cells (WT, dBBLF1, and dBBLF1/R) and their culture media were harvested and separated into cell-free and cell-associated fractions through low-speed centrifugation. The total fraction was prepared without fractionation. Titers of cell-free (B), cell-associated (C), and total virions (D) are shown. (E) The titer of cell-free virions at various time points was monitored. (F) The titer of cell-associated virions at 96 h postreactivation. (G) Observation of the fate of accumulated viruses over a long incubation period. The cell-free viral titers of reactivated HEK293-EBV cells (WT, dBBLF1, and dBBLF1/R) were measured until 192 h. (H) The titer of cell-associated virions at 196 h after lytic induction. The mean ± standard deviation (SD) of three independent biological replicates is shown. Student's *t* test was performed to assess significance. No significant difference (ns), *P* > 0.05; *, *P* < 0.05; **, *P* < 0.01.

To examine the impact of BBLF1 knockout on infectious virus production, we infected EBV-negative cells with virion derived from the culture medium (cell-free virions) and cells (cell-associated virions) separately and monitored viral titers as outlined in Fig. 3A. Cell-free virion production in dBBLF1 was significantly lower than in the WT or dBBLF1/R (Fig. 3B). In contrast, cell-associated virion levels were higher for the knockout virus (Fig. 3C). When virions were collected without fractionation (total virions), no significant difference was observed among cell lines (Fig. 3D). These data suggest that BBLF1 plays a role in the secretion of viruses into the supernatant rather than viral maturation. To examine whether disruption of BBLF1 delayed viral secretion or reduced viral release over time, we collected cell-free samples at various time points. Extracellular virion release was initiated in all clones at 48 h postreactivation and peaked at 72 h (Fig. 3E). The titer of cell-free virions was lower for the knockout than the WT and revertant viruses at 48 and 72 h, but the levels were comparable at 96 h (Fig. 3E). At 96 h after lytic induction, accumulation of cell-associated virions remained prominent (Fig. 3F). Since we hypothesized that BBLF1 knockout may cause a long delay of viral release and that extracellular virion levels may increase after 96 h, we monitored viral titers for a longer period. The amount of dBBLF1 virus released extracellularly decreased in a time-dependent manner after 96 h postreactivation, but the levels were similar to those of WT and dBBRF1/R samples (Fig. 3G). The titer of the cell-associated virus fraction from the dBBLF1 sample remained slightly higher than titers of the WT and revertant viruses at 192 h (Fig. 3H). These data suggest that BBLF1 knockout results in intracellular accumulation of infectious virions, which are released very slowly over time, whereas extracellular virion titers of the WT and revertant strain decrease sharply.

**FIG 4:**
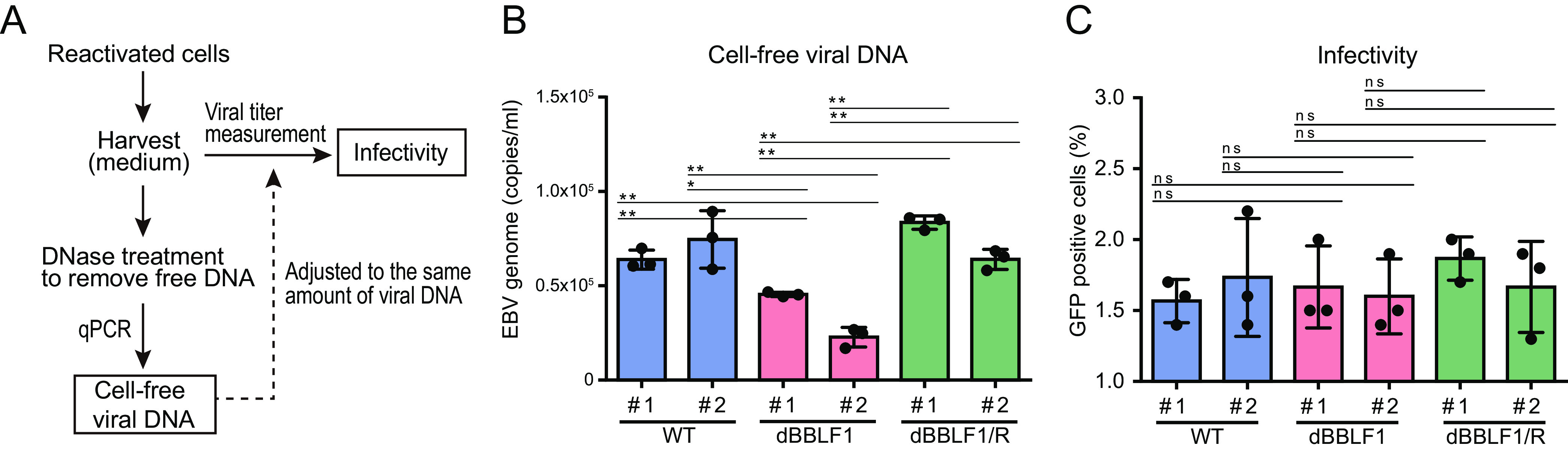
BBLF1 knockout does not affect infectivity. (A) Adjustment of virus fluid based on the amount of virus genome DNA. (B) Measurement of extracellular EBV genome DNA in DNase-treated culture supernatant by qPCR. (C) Infectivity was measured in the presence of equal amounts of virus DNA. Results are means ± SD of three independent biological replicates. Student's *t* test was performed to assess significance. No significant difference (ns), *P* > 0.05; *, *P* < 0.05; **, *P* < 0.01.

To investigate the effect of BBLF1 knockout on infectivity, we measured the cell-free viral DNA level by quantitative PCR (qPCR) and then measured viral titer in the presence of an equal quantity of cell-free viral DNA, as outlined in Fig. 4A. The qPCR results showed a significantly decreased cell-free virus DNA level in dBBLF1 clones (Fig. 4B). By contrast, infectivity was not different among the WT, dBBLF1, and dBBLF1/R clones (Fig. 4C). These data indicate that knockout of BBLF1 does not impact infectivity.

**FIG 5:**
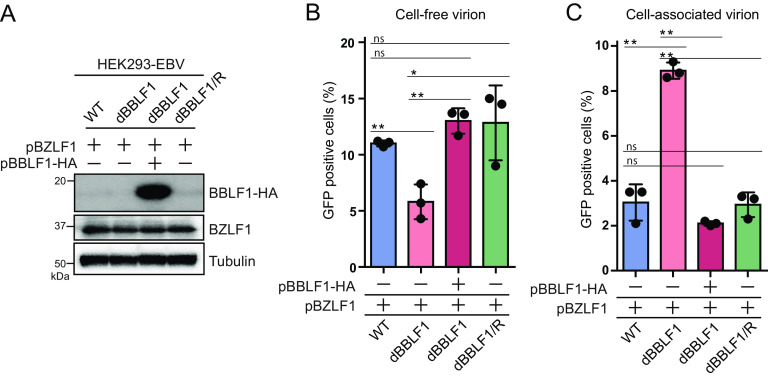
Transcomplementation of BBLF1 in knockout clones rescues the reduction in cell-free virions. BZLF1 expression vectors were expressed in HEK293-EBV WT, dBBLF1, and dBBLF1/R cells. dBBLF1 cells were transcomplemented with BBLF1-HA expression vectors. After 72 h of incubation, samples for cell-free and cell-associated virion titer and immunoblotting were collected. (A) Expression levels of transcomplemented BBLF1-HA, BZLF1, and tubulin obtained by IB using the indicated antibodies. (B) Cell-free virion. (C) Cell-associated virion. Results are means ± SD of three independent biological replicates. Student's *t* test was performed to assess significance. No significant difference (ns), *P* > 0.05; *, *P* < 0.05; **, *P* < 0.01.

We further investigated whether exogenous supply of BBLF1 to dBBLF1 could restore the reduction in virion release during the lytic cycle. Exogenous expression of BBLF1 significantly increased virion release to a level comparable to WT and dBBLF1/R cells (Fig. 5A and B). Overexpression of BBLF1 significantly reduced the number of accumulated virions in BBLF1-knockout cells (Fig. 5C). Taken together, these data indicate that BBLF1 expression during the production phase promotes the release of virions.

### Mature viruses accumulate in the secretory vesicles of knockout virus-infected cells.

**FIG 6:**
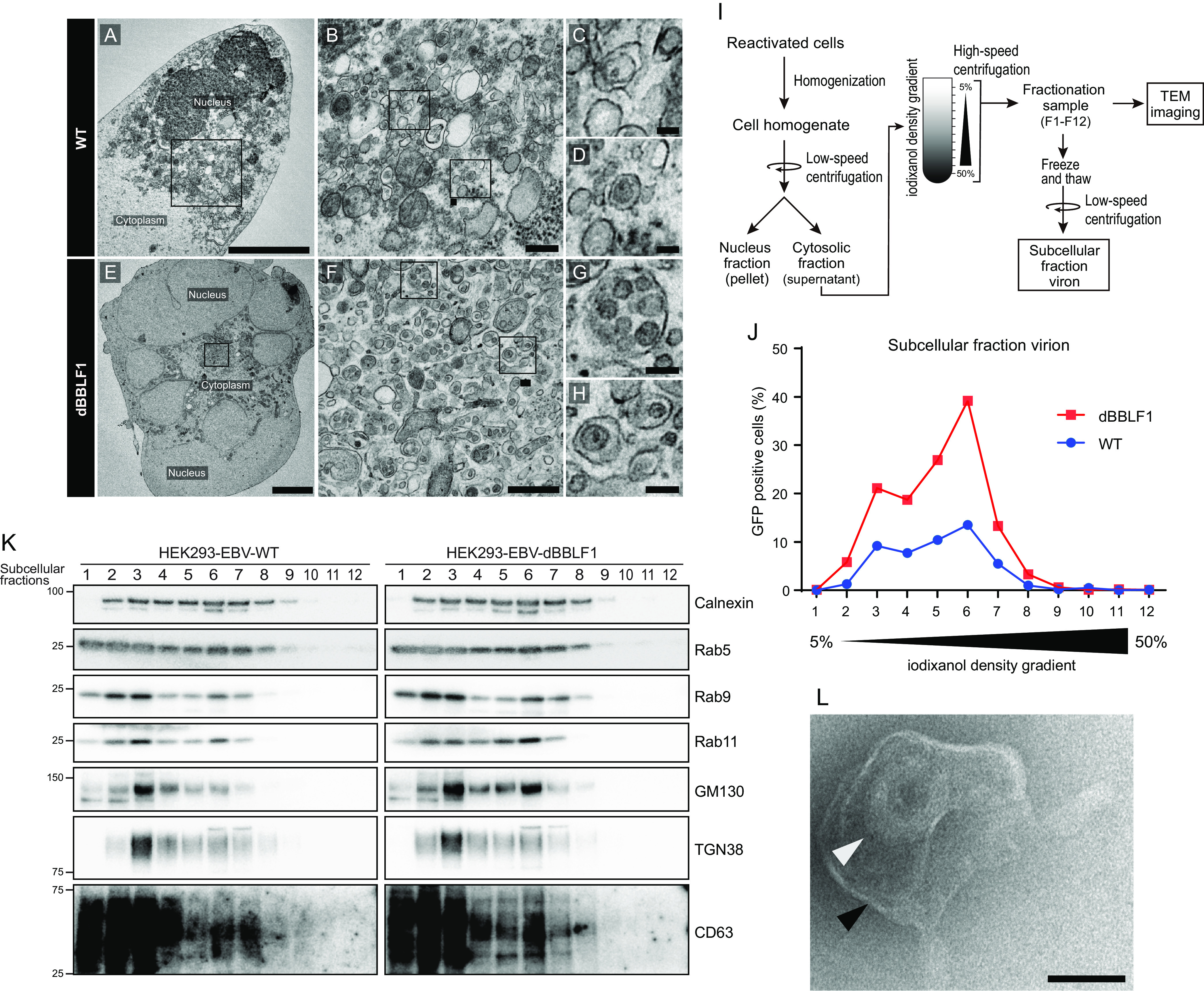
Unreleased virions accumulate in secretory vesicles. Visualization via electron microscopy of the cellular localization of unreleased virions from WT and BBLF1-knockout virus-infected cells. (A) Ultrathin section of a reactivated HEK293-EBV WT cell; scale bar, 5 μm. (B to D) Ultrathin sections of the cytoplasmic area; scale bar, 0.5 μm for panel B and 0.1 μm for panel C. (D) Vesicles harboring single viruses or virus-like particles. (E) Ultrathin section of a reactivated HEK293-dBBLF1 cell; scale bar, 5 μm. (F to H) Ultrathin sections of the cytoplasmic area; scale bar, 0.5 μm for panel F and 0.1 μm for panel G. (H) Vesicles harboring single or multiple viruses or virus-like particles. (I) Diagram of subcellular fractionation of reactivated HEK293-EBV WT and dBBLF1 cell organelles. Reactivated HEK293-EBV cells were harvested at 72 h, disrupted with a Potter-Elvehjem grinder, and centrifuged to remove the nuclear fraction. The cytosolic fraction was poured into an iodixanol (OptiPrep) gradient tube and ultracentrifuged. The resulting fractions (F1 to F12) were collected, aliquoted, frozen, and used for further analysis. (J) The titer of infectious viruses in the subcellular fractions of reactivated HEK293-EBV WT and dBBLF1 cells. (K) Detection of organelle markers in the subcellular fractions. IB was performed using antibodies against calnexin, Rab5, Rab9, Rab11, GM130, TGN38, and CD63. (L) Transmission electron microscopy (TEM) image of a mature virus (white arrow) and a vesicle (black arrow); scale bar, 0.1 μm. The image is of subcellular fraction 6 of dBBLF1 cells.

To determine where BBLF1-knockout viral particles accumulate, we observed lytically induced HEK293-EBV WT and dBBLF1 cells by electron microscopy. Viral particles or virus-like particles accompanying vesicles were observed in both types of cells (Fig. 6A to H); however, more virus particles accumulated in dBBLF1 cells than WT cells. Due to the low number of accumulated viral particles in the WT cells, we hypothesized that BBLF1 knockout affects intracellular trafficking. To test this hypothesis, we identified cellular organelles containing infectious viruses using the technique of cellular organelle fractionation (43). Reactivated cells were mechanically disrupted, and the cytoplasmic fractions containing organelles were separated through iodixanol gradient centrifugation. The fractions (F1 to F12) were collected and analyzed for viral titer, as shown in Fig. 6I. The maximum numbers of virions were detected for both the WT and deficient strains in fraction F6. The infectious viral titer was higher in dBBLF1 than the WT (F3 to F7) (Fig. 6J), possibly because BBLF1 deletion led to intracellular accumulation of progeny virions. To examine the abundance of intracellular organelles in each fraction, we performed immunoblotting analysis targeting organelle marker proteins such as calnexin, Rab5, Rab9, Rab11, GM130, TGN38, and CD63. Band signals associated with Rab9 and Rab11 were frequently detected in fractions F3 and F6, where high levels of infectious viruses were also detected (Fig. 6J and K). Observation of fractions of dBBLF1 containing the largest numbers of infectious viruses by electron microscopy confirmed the presence of virus particles in membranous vesicles (Fig. 6L). This result suggests that BBLF1 deficiency leads to an accumulation of infectious viruses in intracellular organelles involved in intracellular trafficking, including secretory vesicles.

### A cluster of acidic amino acids in BBLF1 is required for optimal virion release.

**FIG 7:**
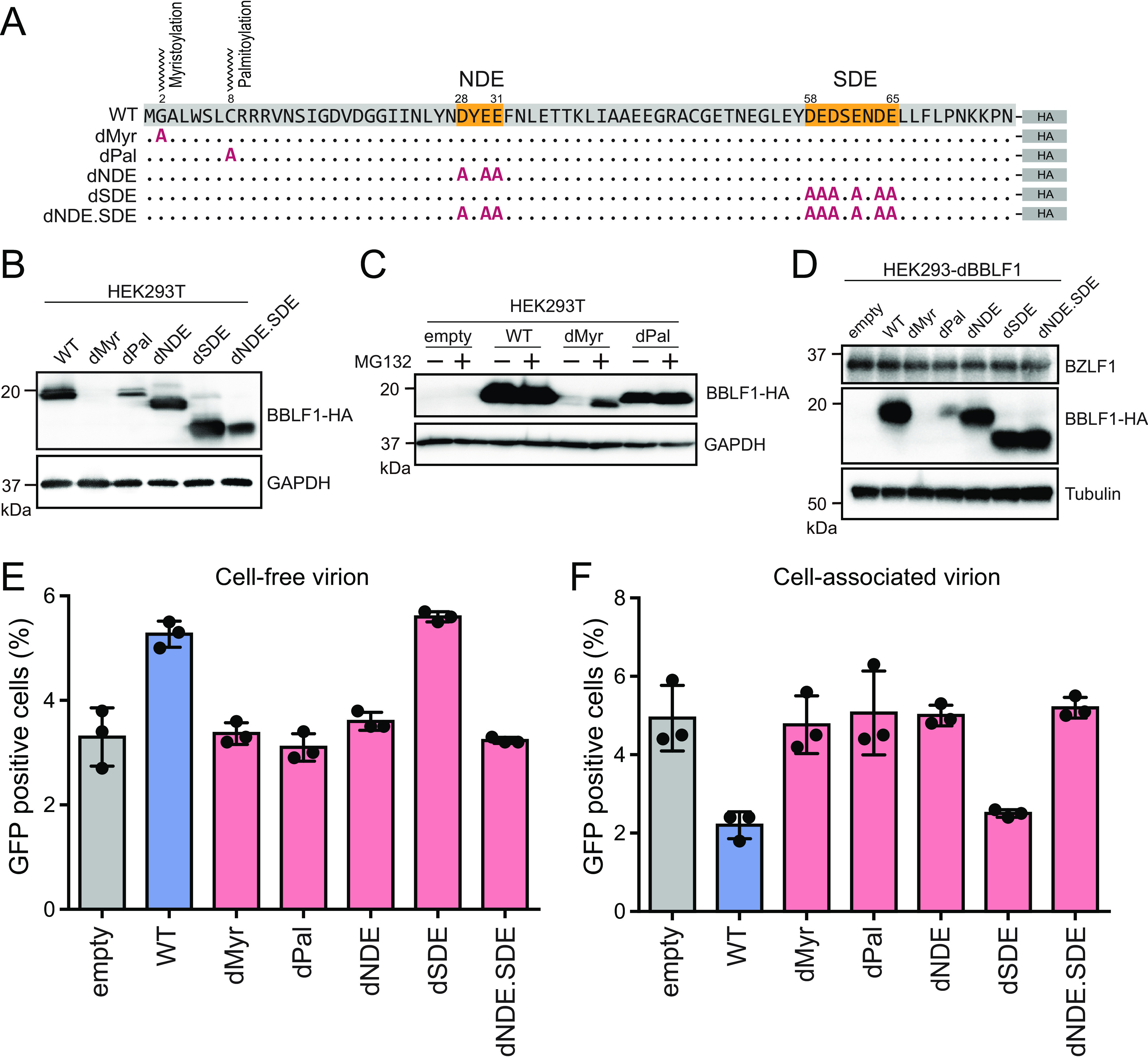
An acidic amino acid cluster is required for virion release. (A) Construction of BBLF1 mutant expression vectors. Through site-directed mutagenesis, alanine substitution mutations were generated at the targeted amino acids. G2A and C8A mutations were generated to obtain BBLF1 myristoylation (Myr) and BBLF1 palmitoylation (Pal) mutants, respectively. The BBLF1 acidic amino acid motifs, NDE (amino acids 28 to 31), SDE (amino acids 58 to 65), and both NDE and SDE, were also substituted with alanine. (B) IB of HEK293T cells transfected with mutant vectors showing the expression of BBLF1 mutants. (C) IB of cells transfected with BBLF1 mutants, with or without the proteasome inhibitor MG132. (D) IB of reactivated HEK293-dBBLF1 cells transcomplemented with mutant vectors showing the expression of BZLF1, BBLF1 mutants, and tubulin. Cell-free (E) and cell-associated (F) infectious viral titer of reactivated HEK293-dBBLF1 cells transcomplemented with the constructed mutant vectors. Results are means ± SD of three independent biological replicates.

BBLF1 is a myristoylated and palmitoylated protein, and it has two acidic clusters of amino acids, NDE (amino acids 28 to 31) and SDE (amino acids 58 to 65) (38). In the context of virion release, we investigated the roles of these posttranslational modifications and acidic motifs in the function of BBLF1. The expression of alanine-substituted BBLF1 mutants, which were constructed as illustrated in Fig. 7A, was determined through immunoblotting. Immunoblotting revealed that the expression levels of dMyr or dPal were reduced compared with those of the WT (Fig. 7B), possibly because the loss of either lipid modification affected the stability of the protein. Treatment with MG132, a proteasome inhibitor, partially rescued the reduction in dMyr expression (Fig. 7C). This result is consistent with a previous report that the stability of BBLF1 requires myristoylation and palmitoylation (38).

To further examine whether those posttranslational modifications and acidic clusters affected viral release, we performed a complementation assay using cells infected with dBBLF1 (Fig. 7D and E). The dMyr and dPal mutants did not restore viral release, possibly due to their reduced levels of protein expression. The dNDE mutant did not change the production level of cell-free virion; conversely, the cell-associated virion of the sample with SDE mutant transfection was elevated unexpectedly to the level of the WT. Interestingly, mutants lacking both NDE and SDE (dNDE.SDE) completely failed to promote viral release (Fig. 7E and F). These results do not contradict previous reports that either the NDE or SDE domain is sufficient for TGN targeting (38) and that interaction of BBLF1 with BGLF2 through the NDE domain is important for efficient progeny production (39). Taken together, these data indicate that acidic amino acid cluster motifs, especially NDE, are crucial to the EBV virion release process.

### The C-terminal region of BBLF1 suppresses virion production.

**FIG 8:**
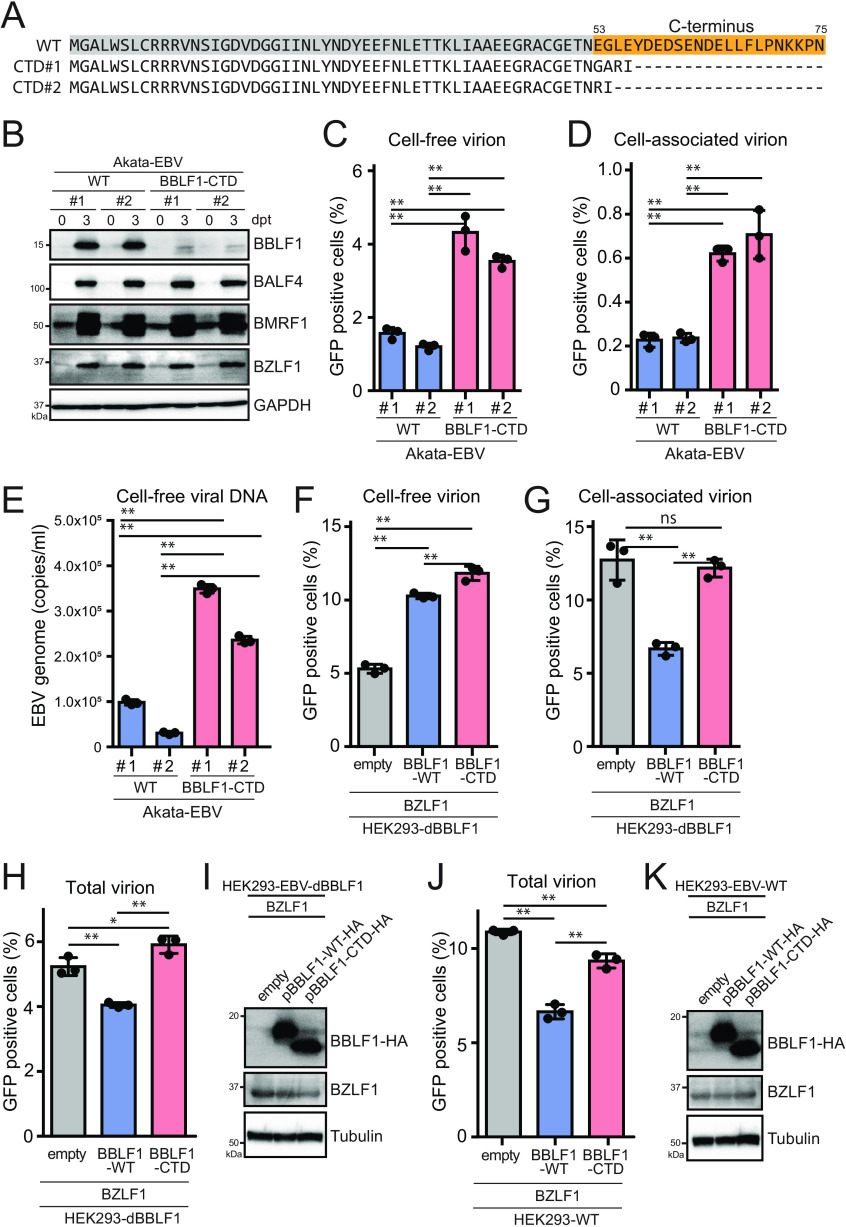
The C-terminal region of BBLF1 can partially suppress virion release. (A) Amino acid sequences of mutants with a BBLF1-C-terminal domain (CTD) mutation obtained through gene editing. (B) Akata-EBV cells were reactivated through treatment with anti-human IgG for 3 days and then subjected to IB to assess the expression of BZLF1, BMRF1, BALF4, and BBLF1. (C, D) Cell-free (C) and cell-associated (D) virion titers of Akata-EBV cells at 3 days postreactivation. Cell-free and cell-associated virion samples were obtained as described in Fig. 3A. (E) EBV genome copies in DNase-treated culture supernatant. (F to H) Transcomplementation of the BBLF1-knockout virus. HEK293-dBBLF1 cells were transfected with BBLF1-HA expression vectors (WT or BBLF1-CTD mutant) during lytic reactivation and their cell-free (F), cell-associated (G), and total (H) progeny virions were quantified. (I) IB of transcomplemented dBBLF1 cells showing the expression of BZLF1, BBLF1-WT-HA, BBLF1-CTD-HA, and tubulin as a housekeeping gene. (J) A similar experiment to that shown in Fig. 8H was conducted, except that WT HEK293-EBV cells were used. (K) IB of transcomplemented WT cells showing the expression of BZLF1, BBLF1-WT-HA, BBLF1-CTD-HA, and tubulin as a housekeeping gene. The data are means ± SD of three independent biological replicates. Student's *t* test was performed to assess significance. No significant difference (ns), *P* > 0.05; *, *P* < 0.05; **, *P* < 0.01.

To extend the previous results obtained using the B95-8 strain of EBV and HEK293 cells, we aimed to prepare a BBLF1-knockout EBV of the Akata strain using the clustered regularly interspaced short palindromic repeats (CRISPR)/Cas9 system. However, knockout of the BBLF1 gene through gene editing was difficult because the BBLF1 gene has an N-terminal overlap with the BBLF1-adjacent BGLF5 gene, which limits the region available for editing. In addition, the expression of the adjacent BGLF3, BGLF3.5, BGLF4, and BGLF5 genes is regulated by a bicistronic or polycistronic translation mechanism, indicating that these proteins are translated from a common mRNA (44, 45). Therefore, we carefully placed guide RNAs in the C-terminal domain (CTD) of the protein. In this manner, we obtained two independent mutant clones of the Akata virus, in which the CTD, including the SDE domain, was disrupted (Fig. 8A). When reactivation was induced in BBLF1-CTD-infected B cells, no BBLF1 protein was detected, as the CTD contains the epitope peptide sequence necessary for antibody production, which likely affected the expression levels of BBLF1 in comparison to that of WT. The expression of BMRF1 phosphorylated by BGLF4 was unchanged, suggesting no effect of C-terminal deletion on the bi/polycistronic translation machinery (Fig. 8B). Unexpectedly, the BBLF1-CTD mutant exhibited increased cell-free and cell-associated virion production compared to that of the WT (Fig. 8C to D). The question thus arose as to whether BBLF1-CTD in Akata cells enhanced virus production or infectivity. To address this question, we measured the cell-free viral DNA levels of WT and CTD clones after DNase treatment. qPCR revealed a significant increase in the cell-free virus DNA level of CTD clones (Fig. 8E). These data indicate that BBLF1-CTD increased virus production.

To validate the finding that C-terminal truncation of BBLF1 resulted in increased production of both cell-free and cell-associated progeny virions, we conducted transcomplementation assays using HEK293-EBV-dBBLF1 cells, which were prepared as shown in Fig. 2. Overexpression of WT BBLF1 in BBLF1-knockout virus-infected cells increased cell-free virion abundance while decreasing cell-associated virion levels (Fig. 8F and G) as expected (see Fig. 7E and F). Exogenous supply of BBLF1-CTD lacking 23 C-terminal amino acids augmented cell-free progeny but did not significantly affect the level of cell-associated virion particles (Fig. 8F and G), resulting in increased total virion levels (Fig. 8H). The exogenous expression of BBLF1-WT and CTD in dBBLF1 cells was determined by immunoblotting (Fig. 8I).

Lastly, we tested HEK293-EBV WT but not HEK293-EBV-dBBLF1 cells. Overexpression of BBLF1-WT in cells infected with WT EBV significantly decreased total progeny production, suggesting that excessive BBLF1 is disadvantageous for EBV reproduction (Fig. 8J, blue bar). An overexpression mutant of BBLF1-CTD also exhibited reduced progeny levels, although the reduction was notably smaller (Fig. 8J and K, red bar) compared to BBLF1-WT overexpression.

From these results, we conclude that the C terminus of BBLF1 has a partial suppressive function in the context of virus production, and thus, the BBLF1 CTD plays a role in regulating BBLF1.

## DISCUSSION

In this study, we identified BBLF1 expression in EBV-infected B cell lines at a late stage of lytic infection. We found that BBLF1 knockout reduced the release of viral particles into the extracellular space, instead leading to the accumulation of mature viral particles in the cytoplasm. Organellar fractionation analysis and electron microscopy revealed that the accumulated virions were located in intracellular organelles, such as the TGN and endosomes, which are rich in secretory vesicles. Furthermore, we found that a cluster of acidic amino acids in BBLF1 regulates virion release. A mutant lacking a C-terminal acidic amino acid cluster (SDE) showed increased efficiency of infectious virus production compared to that of the WT, providing the first example of a tegument protein partially suppressing viral production.

The role of HSV-1 UL11, a homolog of BBLF1, in viral replication is well understood: UL11 knockout resulted in marked reduction of extracellular virion levels (40). The accumulation of nonenveloped nucleocapsids in the cytoplasm in the absence of UL11 indicated that UL11 contributes to acquisition of the envelope. Accumulation of nonenveloped nucleocapsids in UL11-knockout virus-infected cells was also observed within the nuclear membrane, suggesting that UL11 promotes viral transport out of the nucleus (40). In addition to UL11 of HSV-1, UL99 of HCMV and ORF51 of equine herpesvirus type 1 are essential for viral growth and envelope acquisition (40, 41, 46). Moreover, while duck plague virus UL11 and murine gammaherpesvirus 68 (MHV-68) ORF38 are not essential to viral replication, they have been predicted to contribute to viral morphogenesis, as their gene products are packaged within particles during secondary envelopment (47, 48). Based on the common involvement of these homologs in secondary envelopment and the previous finding that BBLF1 is located on the envelope as an outer tegument (29), we hypothesized that BBLF1 facilitates cytoplasmic virion envelopment. One unanticipated result of this study was that BBLF1 knockout reduced extracellular virion abundance, whereas total virion levels, including both the intra- and extracellular fractions, were comparable to the WT (Fig. 3D). Furthermore, electron microscopy images showed no abnormal nucleocapsids in the nuclei of BBLF1-knockout virus-infected cells. Thus, unlike its homologs, BBLF1 affects the transport-to-release phase of infectious virions after acquisition of the final envelope but not nuclear membrane transport or virion maturation.

The mechanism through which BBLF1 facilitates the transport-to-release process remains unclear. Alphaherpesviruses bud on TGN- or endosome-derived vesicular membranes expressing viral glycoproteins (21, 49, 50). In the HCMV protein UL99, named pp28, the N-terminal region, including the site of myristoylation and the acidic amino acid cluster, is sufficient for viral growth, while the C-terminal 2/3 of the protein is dispensable (51). In human herpesvirus 6 (HHV-6), the cytosolic capsid buds into the multivesicular body (MBV) via the ESCORT (endosomal sorting complex required for transport) mechanism (52). MHV-68 has been observed budding into vesicular membranes near the TGN and exocytic vesicles packaged with single or multiple virions (53). The knockdown of three Rab GTPases, Rab8a, Rab10, and Rab11a, promoted viral accumulation and suppressed viral release, indicating that EBV utilizes the host cellular secretory pathway to support progeny virion release (31). We observed that BBLF1 knockout led to an accumulation of budding viral particles in the vesicular membrane, and some viral particles appeared as multivesicular bodies containing numerous internal small vesicles under an electron microscope. Fractionation analysis showed that the fraction containing infectious virus particles was associated with signals of the late endosomal marker, Rab9, and the endosomal recycling marker, Rab11. In addition, the Golgi-associated marker, GM130, and TGN38 were partially correlated with the viral titer (Fig. 6K). We were unable to precisely determine the location of viruses that accumulated via BBLF1 knockout. Further research is required to address whether BBLF1 regulates the TGN-associated exocytosis pathway or exosomal release pathway via the MBV.

BBLF1 is required for virus maturation, but its overexpression can potentially be harmful to virus. Notably, the C terminus of BBLF1 might have an autoinhibitory effect (Fig. 8). Certain viral genes have conflicting functions depending on the intracellular environment and stage of the viral life cycle. For example, EBNA1 is a viral gene with two opposing roles, one of which is associated with latent infection; EBNA1 disruption during latent infection induces spontaneous reactivation, indicating that it suppresses lytic infection. Meanwhile, after reactivation, EBNA1 promotes lytic gene expression, thereby enhancing the efficiency of viral production (9). When and how BBLF1 switches between promotion and suppression of viral production remains unclear, but we predict that BBLF1 negatively regulates virus production during primary infection, thereby increasing the rate of latent infection. Alternatively, BBLF1 may promote an abortive lytic infection to avoid cell death (54). The C-terminal region of BBLF1 has low sequence homology with other herpesviruses and is assumed to represent a survival strategy unique to gammaherpesviruses; further analysis of its effects on the viral life cycle is needed to clarify the biological significance of BBLF1.

In conclusion, we performed initial functional analysis of BBLF1 using BBLF1-knockout viruses and found that BBLF1 contributes to the viral release process. Furthermore, we found that an acidic amino acid cluster enhances the efficiency of viral release. This is the first report showing that the CTD of BBLF1 suppresses viral production, suggesting that the BBLF1 protein plays dual roles. The molecular mechanism through which BBLF1 suppresses viral production remains unclear, and BBLF1 may fine-tune the secretion of progeny virions.

## MATERIALS AND METHODS

### Plasmids and reagents.

The preparation of the pBZLF1, pBBLF1, and pBDLF4 expression vectors has been described previously (17, 55, 56). Several BBLF1 mutant vectors were constructed through inverse PCR using KOD One master mix (TOYOBO) with pBBLF1-HA (hemagglutinin) as the template and the following primers: dBBLF1 forward (ggacggggaattctacccatacgatg) and reverse (tagaattccccgtccacgtcgcctat), dMyr forward (AGAatggCTgccctctggtctctttg) and reverse (gagggcAGccatTCTAGAGGGCCCG), dPal forward (tctcttAgccgacgacgagtcaac) and reverse (tcgtcggcTaagagaccagagggcac), dNDE forward (atgCctatgCggCgtttaacctggagactac) and reverse (aacGccGcatagGcattatacaggttgatta), dSDE forward number 1 (tatgCtgCggCctctgaaaatgatgaattg) and reverse number 1 (agagGccGcaGcatattcgagcccctcgtt), dSDE forward number 2 (tctgCaaatgCtgCattgctgtttttgccaaat) and reverse number 2 (caatGcaGcatttGcagagGccGcaGcatattc), and CTD forward (AAACCAACGAATTCTACCCATACGATGTTCCAG) and reverse (TAGAATTCGTTGGTTTCCCCGCAGGCCCTCCCT). To edit BBLF1 of the Akata strain, Cas9-encoded pX459 (Addgene) carrying a BBLF1-targeting guide RNA was generated through cloning of the following annealed oligonucleotides into the BbsI site of the pX459 vector. The three pairs of oligonucleotides were annealed and inserted into the pX459 vector as follows: sgBBLF1 number 1 forward (CACCCAACCTGTATAATGACTATG) and reverse (AAACCATAGTCATTATACAGGTTG), sgBBLF1 number 2 forward (CACCCTATTAGCTTAGTAGTCTCC) and reverse (AAACGGAGACTACTAAGCTAATAG), and sgBBLF1 number 3 forward (CACCATCATATTCGAGCCCCTCGT) and reverse (AAACACGAGGGGCTCGAATATGAT). The amplified PCR product was introduced into competent E. coli DH5 (Nippon Gene), and clonal cells carrying the plasmid with the optimal sequence were grown for plasmid isolation. The plasmid was isolated using the Plasmid midikit (Qiagen).

The following reagents were used for activation of EBV-infected cells: 12-*O*-tetradecanoylphorbol-13-acetate (Sigma), calcium ionophore (Sigma), sodium butyrate (Wako), and goat anti-human IgG (Jackson Immuno Research Inc.). Phosphonoacetic acid (PAA) (Sigma) was used to inhibit viral DNA replication. Monoclonal antibodies against glyceraldehyde 3-phosphate dehydrogenase (GAPDH), tubulin, HA-tag, calnexin, GM130, Rab5, Rab9, Rab11, and TGN38 were purchased from Cell Signaling Technology. An antibody against CD63 (Ts63) was obtained from Thermo. Antibodies against BZLF1, BMRF1, BALF4, BDLF4, and BALF2 were used as described previously (29). Rabbits were immunized with the BBLF1 polypeptide (C+LEYDEDSEND) conjugated to keyhole limpet hemocyanin (KLH), and affinity-purified polyclonal anti-BBLF1 antibodies were obtained from serum.

### Cell lines and lytic reactivation for virus production.

HEK293, HEK293T, and HEK293 cells carrying EBV-BAC as an episome (HEK293-EBV) were cultured in Dulbecco's modified Eagle’s medium (DMEM) (Sigma) supplemented with 100 U/mL penicillin-streptomycin (Gibco) and 10% fetal bovine serum (FBS). Akata EBV-positive and EBV-negative cells, EBV-infected AGS cells (AGS-EBV), and B95-8 cells were cultured in RPMI 1640 medium (Sigma) supplemented with 100 U/mL penicillin-streptomycin and 10% FBS. EBV-infected cell lines including B95-8, Akata-EBV, AGS-EBV, and HEK293-EBV were lytically reactivated for viral production and further analysis. Culture supernatants and cells were generally harvested at 48 to 72 h after lytic reactivation. AGS-EBV cells maintained in 400 μg/mL G418 were transfected with the pBZLF1 vector using the Neon electroporation system (Thermo). HEK293-EBV cells were propagated in DMEM containing 150 μg/mL hygromycin B (TaKaRa) and reactivated via pBZLF1 transfection using Lipofectamine 2000 (Thermo). Goat anti-human IgG was used to reactivate EBV-positive Akata cells maintained in 750 μg/mL G418. B95-8 cells were treated with TAB [T, 12-*O*-tetradecanoylphorbol-13-acetate (20 ng/mL); A, calcium ionophore (1 μM); B, sodium butyrate (5 mM)] for reactivation and PAA (400 μg/mL) to inhibit viral DNA replication.

### Immunoblotting.

HEK293T cells were transfected with the designated plasmids using Lipofectamine 2000 (Thermo) for assessment of protein expression. Alternatively, in some cases, EBV-carrying clonal cells were lytically reactivated through targeted methods and harvested at the indicated time points for immunoblotting. To prepare a solubilized protein sample, harvested cells were washed in phosphate-buffered saline (PBS) and lysed in lysis sample buffer (50 mM Tris-HCl, 2% sodium dodecyl sulfate, 10% glycerol, 6% 2-mercaptoethanol, and 0.0025% bromophenol blue; pH 6.8). Equal amounts of proteins were resolved through electrophoresis on a sodium dodecyl sulfate-polyacrylamide gel (SuperSep Ace; Wako), transferred onto polyvinylidene difluoride membranes (Millipore), reacted with designated primary antibodies, and then reacted with horseradish peroxidase (HRP)-linked anti-rabbit or anti-mouse IgG (Cell Signaling Technology) as secondary antibodies. Proteins were visualized through reaction with an HRP substrate, namely, Forte (Merck) or Chemi-Lumi One Ultra (Nacalai Tesque, Inc.). Luminescent signals were detected and analyzed using the LuminoGraph II EM imaging system (ATTO).

### Gene recombination of EBV-BAC DNA.

BBLF1-deficient EBV was generated from EBV-BAC, a BAC containing the cloned EBV genome, following the homologous recombination technique (55). The EBV-BAC carrying the genome of the B95-8 strain was provided by Wolfgang Hammerschmidt (57). The WT EBV-BAC was first transfected and maintained in Escherichia coli. During the homologous recombination of EBV-BAC in E. coli, a cassette of neomycin resistance and streptomycin sensitivity (Neo^s^/St^r^) genes was inserted into the BBLF1 sequence, resulting in intermediate-1; this was later replaced with the BBLF1-nonsense sequence, producing BBLF1-deficient EBV-BAC (dBBLF1). Similarly, after the generation of intermediate-2 with the Neo/St cassette from dBBLF1, a revertant EBV-BAC was constructed that carries the same sequence as WT BBLF1 (dBBLF1/R). PCR products were purified using a PCR product extraction kit (Qiagen), and EBV-BAC was extracted with the NucleoBond BAC 100 kit (TaKaRa). A Gene Pulser III (Bio-Rad) instrument was used for transfection of EBV-BAC and PCR products. The extracted EBV-BAC was digested using EcoRI or BamHI restriction enzymes and visualized through 0.6% agarose gel electrophoresis. Sequencing was conducted to confirm that the targeted manipulation of the EBV-BAC was successful. The isolated EBV-BAC was transfected into HEK293 cells using Lipofectamine 2000 and selected using hygromycin B for 2 weeks. Finally, hygromycin-resistant and green fluorescent protein (GFP)-positive cell colonies were selected and HEK293-EBV clonal lines were established. Individual PCR products were obtained through PCRs, using the rpsL-neo vector (Gene Bridges) as a template and the following primers: for BBLF1 Neo/St, 5′- ATGGGTGCCCTCTGGTCTCTTTGCCGACGACGAGTCAACTCCATAGGCGACGTGGACGGGggcctggtgatgatggcgggatc-3′ and 5′-TCGGCCGCTATTAGCTTAGTAGTCTCCAGGTTAAACTCCTCATAGTCATTATACAGGTTGtcagaagaactcgtcaagaagg-3′. To construct BBLF1 containing an antisense sequence, a vector carrying a nonsense mutation in BBLF1 was first constructed through inverse PCR using the following primers: 5′-ggacgggTgaataatcaacctgtata-3′ and 5′-attattcAcccgtccacgtcgcctat-3′. The PCR product was amplified using a vector.

### CRISPR/Cas9-edited EBV mutant.

The generation of EBV mutants through CRISPR/Cas9-based editing has been described previously (58). Briefly, AGS-EBV cells carrying the recombinant EBV genome (Akata strain) pX459-BBLF1 were transfected using Lipofectamine 2000 and selected with puromycin. Then, lytic reactivation was conducted as described above; eventually, viruses were produced. The cell-free culture supernatant was used to infect Akata (−) cells, and a limiting dilution assay was used for selection of GFP-positive and G418-resistant clonal cells. G418 was provided in the medium at a concentration of 750 μg/mL until 3 to 4 weeks postinfection.

### Infectious viral titer.

Reactivated HEK293-EBV or Akata-EBV cells were harvested after a specified incubation period along with the spent culture medium and centrifuged at low speed; the supernatant contained cell-free virions, while cell-associated virions were obtained from the cell pellets after further processing. The cell pellets were frozen, thawed, and resuspended in medium, and cell debris was then removed from the medium through high-speed centrifugation to obtain cell-associated virions. For collection of total virions including both the cell-free and cell-associated fractions, harvested cells and spent media were not initially separated; instead, they were frozen, thawed, vortexed, and centrifuged, after which the supernatant was collected. To determine viral titer, 1 × 10^6^ EBV-negative Akata cells were cocultured with 1 mL viral medium using a tube rotator for 2.5 h, after which the medium was replaced with fresh medium and incubation was continued. At 48 h postinfection, cells were fixed with 4% paraformaldehyde, and the frequency of GFP-positive cells was examined by flow cytometry (Gallios; Beckman Coulter).

### Quantification of cell-free virus DNA.

The number of virus particles released into culture medium from lytically induced cells was quantified by enumerating viral genome copies as reported previously (59). Briefly, 200 μL of culture supernatant was pretreated with Turbo DNase I (Invitrogen) to remove free DNA. Viral genome intact inside the virus particle was isolated using the DNeasy blood and tissue kit (Qiagen) according to the manufacturer’s instructions. Next, the number of EBV genomes was quantified by qPCR using Fast Start Universal Probe Master (Rox; Roche Applied Science) and the isolated viral genome as the template. A standard curve was generated using serial dilutions of a standard solution of EBV genome. The primers and probes were designed to target the BALF5-coding region of EBV.

### Electron microscopy.

HEK293-EBV cells were lytically reactivated, harvested after 3 days, washed in PBS, and fixed with 2% glutaraldehyde. Secondary fixation was conducted with 1% OsO_4_, followed by treatment with 1% uranyl acetate. For dehydration, the cells were incubated in a series of ethanol solutions with concentrations increasing gradually from 70% to 100%. This was followed by the embedding of cells in resin, thin sectioning, mounting of the slices on a specimen grid, and staining with uranyl acetate and lead citrate. Transmission electron microscopy (TEM) images were captured using a JEOL JEM-1400 PLUS transmission electron microscope.

### Subcellular fractionation.

Reactivated HEK293-EBV cells (2.5 × 10^7^) were harvested, resuspended in homogenization medium (0.25 M sucrose, 140 mM NaCl, 1 mM EDTA, 20 mM Tris-HCl, pH 8.0), and homogenized on ice with a Potter-Elvehjem grinder. To obtain the cytoplasmic fraction, the cell suspension was centrifuged at 1,000 × *g*, and the pellet was removed as the nuclear fraction. A continuous 5 to 50% (wt/vol) iodixanol (OptiPrep) gradient was prepared using Gradient Master (BioComp). Next, the cytosolic fraction was poured into the tube as a top layer. The gradient tubes were ultracentrifuged in an Optima XPN-80 (Beckman) centrifuge at 48,000 × *g* for 18 h. After ultracentrifugation, the gradient solution was collected from top to bottom as 12 fractions. For infectious viral titer measurement, fractions were frozen and thawed, and cell debris was then removed through centrifugation. After Akata cells were infected, each fraction was adjusted to a 1-mL volume through the addition of RPMI medium. Lysis sample buffer was added to the fractioned sample, followed by immunoblotting with the designated antibodies to check for the presence of cellular organelle markers. Fractionated samples were placed on hydrophilized grids (Nisshin EM), reacted with 1% uranyl acetate for 10 s, dried, and examined under an electron microscope.
